# Suizidprävention für LGBTQ+-Jugendliche: Notwendigkeit, Modell und Zugänge

**DOI:** 10.1007/s11553-023-01096-7

**Published:** 2024-01-11

**Authors:** Andreas Pfister

**Affiliations:** Institut für Public Health, ZHAW Zürcher Hochschule für Angewandte Wissenschaften, Katharina-Sulzer-Platz 9, Postfach, 8401 Winterthur, Schweiz

**Keywords:** Psychische Gesundheit, Sexuelle Orientierung, Sexuelle und geschlechtliche Minoritäten, Intersektionalität, Geschlechtsidentität, Mental health, Sexual orientation, Sexual and gender minority youth, Intersectionality, Gender identity

## Abstract

**Hintergrund:**

Lesbische, schwule („gay“), bisexuelle, trans und queere Jugendliche („LGBTQ+“) sind stärker gefährdet als ihre heterosexuellen und cis Altersgenoss:innen, mindestens einmal in ihrem Leben einen Suizidversuch zu begehen. Evidenzbasierte suizidpräventive Zugänge und Modelle für LGBTQ+-Jugendliche fehlen jedoch.

**Methodik:**

Anhand deutschsprachiger und internationaler (Forschungs‑)Literatur werden die Hintergründe suizidalen Verhaltens bei LGBTQ+-Jugendlichen aufgezeigt, Lücken identifiziert und basierend darauf ein Modell intersektionaler und multisektoraler Suizidprävention entworfen.

**Ergebnisse:**

Die wenigen Vorschläge und Konzepte zur Stärkung psychischer Gesundheit und Suizidprävention bei LGBTQ+-Jugendlichen stammen aus der Sozialen Arbeit, der Klinischen Psychologie und der Psychiatrie/Medizin. Eine konsequent multisektorale Sichtweise und die Berücksichtigung der Verschränkung weiterer Diversitätsdimensionen wie etwa „race“, sozioökonomischer Status etc. mit sexueller Orientierung und/oder Geschlechtsidentität stehen in der Suizidprävention jedoch aus. Ausgehend vom Modell von Russell und Fish (2016) wurden ein neues konzeptionelles Modell der Suizidprävention für LGBTQ+-Jugendliche entworfen und universelle, selektive und indizierte Zugänge exemplarisch dargelegt.

**Schlussfolgerung:**

Das vorgeschlagene Modell bietet einen neuen konzeptionellen Rahmen, suizidpräventive Maßnahmen auf verschiedenen Ebenen zu verorten, zu planen und durchzuführen, dies intersektional und über verschiedene Professionen und Sektoren hinweg.

## Abstract (en)

**Background:**

Lesbian, gay, bisexual, transgender and queer youth (LGBTQ+) are at a greater risk of attempting suicide at least once in their lives than their heterosexual and cisgender peers. However, evidence-based suicide prevention approaches and models for LGBTQ+ youth are lacking.

**Methods:**

Using German-language and international literature, the background of suicidal behaviour among LGBTQ+ youth was highlighted, gaps were identified and, based on this, a model of intersectional and multisectoral suicide prevention was designed.

**Results:**

The few concepts for strengthening mental health and suicide prevention among LGBTQ+ youth stem from social work, clinical psychology and psychiatry/medicine. However, a consistently multisectoral perspective and consideration of the intertwining of further diversity dimensions such as “race”, socioeconomic status, etc. with sexual orientation and/or gender identity is lacking in suicide prevention. Based on Russell and Fish’s (2016) model, a new conceptual model of suicide prevention for LGBTQ+ youth was designed, and universal, selective and indicated approaches were exemplified.

**Conclusion:**

The proposed model provides a new conceptual framework to locate, plan and implement suicide prevention interventions at different levels, intersectionally and across different professions and sectors.

## Hintergrund

Der internationale Forschungsstand zeigt: Lesbische, schwule [„gay“], bisexuelle, trans und queere Personen (kurz „LGBTQ+-Personen“; s. häufig verwendete Abkürzungen in Tab. 1) sind stärker von suizidalem Verhalten (Suizidgedanken, Suizidversuche) betroffen als die heterosexuelle und cisgender Bevölkerung [12, 14, 16, 17, 27, 32, 34, 39, 45]. Über alle Altersgruppen und LGBTQ+-Personen hinweg muss davon ausgegangen werden, dass die Suizidversuchsrate (Lebenszeitprävalenz) gegenüber der Allgemeinbevölkerung in der Schweiz etwa 4‑mal höher ist und ein Großteil der Suizidversuche im Jugendalter und jungen Erwachsenenalter stattfinden [17]. Das Kindes- und Jugendalter und junge Erwachsenenalter sind deshalb entscheidende Fenster für die Suizidprävention bei LGBTQ+-Personen.

**Tab. 1:** Überblick Abkürzungen. (Eigene Darstellung)

Abkürzung	Bedeutung	Dimension
L	Lesbisch	Sexuelle Orientierung
G	Gay (engl.): schwul	
B	Bisexuell: Frauen wie Männern zugeneigt	
T	Trans oder Transgender (engl.): sich einem anderen Geschlecht zugehörig fühlen als das, mit dem man geboren wurde bzw. das einem bei Geburt zugewiesen wurde, non-binär: nicht nur Frau oder nur Mann; Verortung außerhalb der Binarität Mann-Frau	Geschlechtsidentität
Cis	Cis oder Cisgender (engl.): sich dem Geschlecht zugehörig fühlen, mit dem man geboren wurde bzw. das einem bei Geburt zugewiesen wurde	
Q	Queer (engl.): Überbegriff zur Bezeichnung nicht der Mehrheit entsprechender sexueller Orientierungen und Geschlechtsidentitäten, Questioning (engl.): auf der Suche nach der geschlechtlichen Identität oder/und sexuellen Orientierung	Sexuelle Orientierung oder Geschlechtsidentität
I	Intergeschlechtlich: biologische Merkmale, die nicht eindeutig männlich oder weiblich sind	Geschlechtsmerkmale
+	Das „+“ im englischen Akronym signalisiert, dass weitere Selbst- und Fremdbezeichnungen für die Zugehörigkeit zu sexuellen und geschlechtlichen Minoritäten verwendet werden und keine vollständige Aufzählung möglich ist	Sexuelle Orientierung oder Geschlechtsidentität

Das erhöhte Risiko für suizidales Verhalten kommt nicht direkt durch die sexuelle Orientierung oder die Geschlechtsidentität, sondern vielmehr durch indirekte Faktoren zustande. Zusätzlich zu anderen möglichen generellen Stressoren, die alle Jugendlichen haben können, wie etwa Schulstress, sind LGBTQ+-Jugendliche einem Minoritätenstress ausgesetzt [24]. Darunter fallen externe Faktoren wie Stigmatisierung, Diskriminierung oder gar Gewalt, die LGBTQ+-Jugendliche seitens der Gesellschaft, von Gruppen oder durch andere Individuen erleben können. Interne Faktoren sind individuelles Erwarten von Ab- und Zurückweisung, das Verstecken und Verbergen der eigenen sexuellen Orientierung bzw. Geschlechtsidentität oder verinnerlichte Homo- Bi- bzw. Transnegativität. Diese und weitere Risikofaktoren wurden auch in der empirischen Literatur festgestellt [14, 16, 45]: gegenüber LGBTQ+-Personen abwertende gesellschaftliche Normen und Strukturen, Homo‑, Bi- und Transnegativität [22, 40], Mobbing in der Schule [1, 3, 28, 30, 41, 44], fehlende Akzeptanz der sexuellen Orientierung oder Geschlechtsidentität durch die Familie und das soziale Umfeld [26, 35, 36], Geschlechtsrollennonkonformität [31], geringe Selbstakzeptanz bzw. verinnerlichte Homonegativität [35], psychische Störungen [9], Substanzmissbrauch [23], Schwierigkeiten, über Gefühle oder die eigene sexuelle Orientierung zu sprechen sowie andere Lebenskrisen [22]. Zu den protektiven Faktoren sind unterstützendes Schulklima, akzeptierende und unterstützende Familien wie auch eine LGBTQ+-freundliche Gesundheitsversorgung zu zählen [8, 10, 21, 25, 38]. An diese schließen auch suizidpräventive Bemühungen an.

## Methodisches Vorgehen

**Abb. 1:**
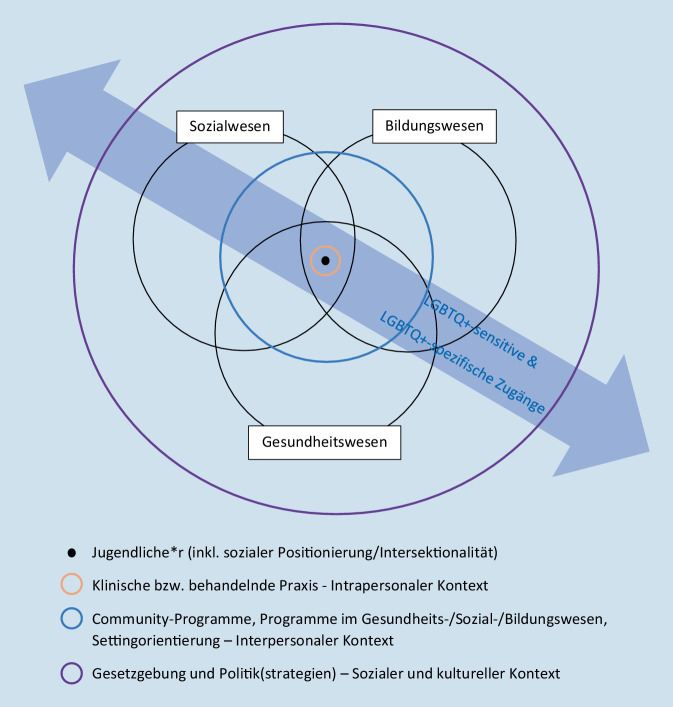
Modell multisektoraler, interprofessioneller und intersektionaler Suizidprävention für LGBTQ+-Jugendliche. (Eigene Darstellung, entwickelt ausgehend von [33])

Der Autor hat 2019 das wissenschaftlich vorhandene Wissen zu den Hintergründen von Suizidversuchen bei LGBTQ+-Jugendlichen und jungen Erwachsenen international gesichtet und basierend auf den identifizierten Forschungslücken ein qualitativ-empirisches Forschungsvorhaben zuhanden des Schweizerischen Nationalfonds formuliert. Das Forschungsprojekt „Suizidversuche von LGBT-Jugendlichen und jungen Erwachsenen in der Schweiz – Kontexte und Hilfesuchverhalten“ (Grant Nr. 192684) wurde 2020 genehmigt und läuft noch bis Ende 2024. Die Literatursuche zur Formulierung des Forschungsantrags erfolgte vorwiegend über die Suchmaschine „Web of Science“ inklusive Einbezug von grauer Literatur und Literatur, die im Schneeballprinzip über in Artikeln verzeichneten Quellen eruiert wurde. Die Suche richtete sich danach, die Prävalenzen suizidalen Verhaltens (insbesondere Suizidversuche) bei LGBTQ+-Jugendlichen und jungen Erwachsenen, mögliche Erklärungsansätze und -modelle und bestehende evidenzbasierte suizidpräventive Zugänge zu identifizieren. Einige Ergebnisse daraus, auch ergänzt um aktuellere Literatur, wurden vorangehend berichtet. Mittels dieser Literatursuche – und in einem anderen vom Autor kogeleiteten nationalen Forschungsprojekt zur Gesundheit von LGBT-Personen [17] inklusive eines narrativen Reviews im Jahr 2020 (s. Suchsystematik im Anhang von [18]) – wurde deutlich, dass nicht nur Forschungslücken zu einem besseren Verständnis des Prozesses hin zu Suizidversuchen bei LGBTQ+-Jugendlichen bestehen, sondern auch keine evidenzbasierten, gut evaluierten suizidpräventiven Zugänge für diese Gruppen vorliegen. Dies steht im Kontrast zum hohen Bedarf an solchen Maßnahmen und dem auch seitens Praxisakteur:innen aus dem Sozial‑, Gesundheits- und Bildungswesen an den Autor herangetragenen Wunsch, trotz fehlender (evaluierter) Programme und Interventionsmodelle doch zumindest auf theoretischer wie empirischer wissenschaftlicher Literatur basierend erste Ansätze zu präsentieren, welche suizidpräventiven Zugänge für LGBTQ+-Jugendliche allenfalls gewinnbringend sein könnten. Unter Einbezug der recherchierten Literatur, aktueller Leitlinien der WHO zur Implementierung von Suizidprävention [42, 43], der Berücksichtigung der Bedeutung intersektionaler und diversitätsgerechter Zugänge in Public Health [2] und der Nutzung und Weiterentwicklung des „conceptual model of contextual influences on lesbian, gay, bisexual, and transgender (LGBT) youth mental health and associated implications for policies, programs, and practice“ [33] wurde ein Modell der Suizidprävention für LGBTQ+-Jugendliche erstellt (s. Abb. 1).

### Evidenzbasierte Zugänge fehlen

Die Literaturrecherche zeigte: Evidenzbasierte, gut evaluierte Zugänge der Arbeit mit LGBTQ+-Jugendlichen, selbst über ganz verschiedene Settings wie Schulen, Community-basierte Organisationen oder die klinischen Behandlung betrachtet, fehlen [5, 33, 37]. Spezifische und LGBTQ+-sensitive Zugänge und Interventionen für LGBTQ+-Jugendliche müssen dringend geschaffen und beforscht werden [5]. Nur so kann eine auf empirische Forschungsresultate abgestützte und wirksame Suizidprävention für und mit diesen Gruppen etabliert werden. Bestehende Vorschläge zur Stärkung psychischer Gesundheit und Suizidprävention bei LGBTQ+-Jugendlichen sind mehrheitlich aus der (sozial)pädagogischen und klinisch-psychologischen/psychiatrischen Perspektive verfasst [14, 20, 33]. Bei allen rücken das Sozial- und Gesundheitswesen in seiner ganzen Breite (u. a. Sozialarbeitende, Kinderärzt:innen, Pflegefachpersonen etc.) und die auch von der WHO vielgeforderte multisektorale Zusammenarbeit [42, 43] nicht in den Blick. Weiter ist zu bemängeln, dass der Diversität außerhalb der Dimensionen Geschlechtsidentität und sexuelle Orientierung noch zu wenig Rechnung getragen wird, die Schnittstellen zu anderen (Benachteiligungs‑)Merkmalen wie z. B. sozioökonomischer Status oder „race“ etc. außenvorbleiben. Eine intersektionale Perspektive fehlt. Ausgehend vom Modell von Russell und Fish [33] wird deshalb nachfolgend der Versuch unternommen, suizidpräventive Zugänge für LGBTQ+-Jugendliche konsequent multisektoral, interprofessionell und intersektional zu denken (s. Abb. 1).

## Multisektorale, interprofessionelle und intersektionale Suizidprävention für LGBTQ+-Jugendliche

Im Zentrum des Modells (s. Abb. 1) steht die jugendliche Person (schwarzer Punkt) inklusive ihrer sozialen Positionierung aufgrund von Merkmalen wie etwa Geschlechtsidentität, sexuelle Orientierung, „race“, sozioökonomischer Status, Beeinträchtigung. Intersektionalität meint dabei, dass sich mehrere soziale Kategorien (z. B. Gender, ethnische Herkunft, sozioökonomischer Status etc.) auf der Ebene des Individuums überschneiden und auf der Makroebene vorhandene ineinandergreifende Systeme von Privilegien und Unterdrückung widerspiegeln bzw. darauf verweisen (z. B. Rassismus, Sexismus, Homo- und Transnegativität; [4]). Eine 15-jährige lesbische weiße cisgender Jugendliche mit hohem sozioökonomischem Status kann etwa auf andere Barrieren, Ressourcen und Reaktionen ihres Umfelds stoßen, als eine 13-jährige Schwarze trans Jugendliche mit niedrigem sozioökonomischem Status, die ohne Hilfe von medizinischem Personal eine männliche Pubertät durchmachen muss und den Geschlechtseintrag in vielen Ländern der Welt noch immer nicht eigenständig und unkompliziert amtlich ändern lassen kann.

Das Individuum ist eingebettet in Systeme des Bildungs‑, Sozial- und Gesundheitswesens (s. schwarze beschriftete Kreise). Das Modell könnte um weitere Systeme und Sektoren wie etwa die Sicherheit/Polizei erweitert werden. Die farbigen Kreise in hellrot, hellblau und lila – von innen nach außen betrachtet – symbolisieren die suizidpräventiven Praxisbereiche und in welchem Kontext diese stattfinden. Der hellblaue große Doppelpfeil, der alle Sektoren und Kontexte von links oben nach rechts unten durchzieht, symbolisiert die Notwendigkeit und Gleichzeitigkeit von LGBTQ+-sensitiven wie auch LGBTQ+-spezifischen suizidpräventiven Zugängen über alle Bereiche und Kontexte hinweg. Kurz: Alle Sektoren und alle Professionen müssen multisektoral in der Regelversorgung ihre Angebote und Zugänge LGBTQ+-inklusiv ausrichten, sensitiv und sensibilisiert für die Bedürfnisse und die Lebenswelten von LGBTQ+-Personen sein. Zugleich sind aufgrund der höheren Gefährdung für suizidales Verhalten LGBTQ+-spezifische Zugänge angezeigt, Zugänge also, die sich gezielt an LGBTQ+-Personen und/oder ihr Umfeld richten.

Der hellrote Kreis nahe am Individuum ist die klinische bzw. behandelnde Praxis in Bezug auf das Individuum (intrapersonaler Kontext). Im Unterschied zu Russell und Fish [33] sind im vorliegenden Modell nicht ausschließlich klinisch-psychologische oder psychiatrische Behandlungen durch entsprechende Fachkräfte (Psycholog:innen, Psychiater:innen) und der Themenkomplex der psychischen Probleme gemeint. Vielmehr handelt es sich um alle biopsychosozialen Probleme eines Individuums, die von einem oder mehrerer Systeme (z. B. Bildung, Soziales, Gesundheit) und der darin tätigen Professionellen „behandelt“ bzw. bearbeitet werden. Es ist der Bereich, wenn sich Probleme beim Individuum bereits mehr oder weniger schwerwiegend manifestiert haben. Die jugendliche Person hat z. B. Probleme in der Schule, ist im Klassenverbund schlecht integriert und wird aufgrund ihres gendernonkonformen Auftretens regelmäßig schikaniert. Sie befindet sich in schulpsychologischer Abklärung, nimmt aufgrund von Unwohlsein und Bauchbeschwerden regelmäßig Termine bei ihrer Hausärztin wahr, befindet sich aufgrund von depressiven Episoden in der Behandlung bei einem Kinder- und Jugendpsychiater und wohnt wochentags in einer sozialpädagogischen Wohngemeinschaft. Da die elterliche Obhut aufgrund von Problemen der Eltern nicht vollumfänglich durch die Eltern wahrgenommen werden kann, ist die zuständige Kindes- und Erwachsenenschutzbehörde involviert. Gesundheitsförderlich und suizidpräventiv Handeln würde in diesem Fall bedeuten, dass alle Sektoren und Professionen (übergreifend!) in der Lage sind, LGBTQ+-sensitive bzw. -spezifische Zugänge einzusetzen. Wenn etwa bekannt ist, dass es sich um eine:n Jugendliche:n mit non-binärer Geschlechtsidentität handelt, der:die sich nicht im binären Spektrum Mann-Frau verortet, können LGBTQ+-spezifische Zugänge greifen. Alle Sektoren und Professionen müssen in der Lage sein, die gesellschaftlichen, sozialen und institutionellen Barrieren, die sich dieser jugendlichen Person stellen, bestmöglich zu erkennen und abzubauen – z. B. Verwendung korrekter bzw. keiner Pronomen, Entgegenwirken von Ausgrenzungsprozessen im Alltag, Anwenden einer adäquaten Hilfe‑/Behandlungsstrategie etc. – und sollten hinsichtlich der höheren Gefährdung suizidalen Verhaltens sensibilisiert sein. Ein besonderer Fokus muss auf die Stärkung der Ressourcen und Lebenskompetenzen gelegt und das Individuum muss in seiner ganzen Mehrdimensionalität betrachtet werden. Eine zu ausschließliche Rahmung des:der Jugendlichen unter dem Label „non-binär“ ist nicht sinnvoll.

LGBTQ+-sensitive bzw. -inklusive Zugänge können und sollen in jedem Fall und in jeder Situation greifen. Auch wenn z. B. nicht bekannt ist, ob es sich um eine:n LGBTQ+-Jugendliche:n handelt, können und sollen die verschiedenen Professionen Offenheit und reflektierte Zugänge gegenüber sexueller und geschlechtlicher Vielfalt aktiv signalisieren. So können sich LGBTQ+-Jugendliche, die noch nicht geoutet sind, leichter öffnen und ihrem Umfeld mitteilen. Auch heterosexuelle und cis Jugendliche, die z. B. aufgrund gendernonkonformen Auftretens schikaniert und/oder sozial als LGBTQ+ gelesen werden, können damit qualifiziert unterstützt werden. Das Eintreten für geschlechtliche und sexuelle Diversität ist kein Randgruppenthema. Es sind potenziell alle Kinder und Jugendlichen von homo-, bi- und transnegativer Ausgrenzung, Diskriminierung oder gar manifester Gewalt betroffen. Dies ist neben vielen anderen ein Grund mehr, weshalb sich die ganze Gesellschaft und auch alle Professionellen – unabhängig ihrer eigenen sexuellen Orientierung oder Geschlechtsidentität – dafür einsetzen sollten, auch in klinisch-therapeutischen, problembehandelnden und präventiven Kontexten im Bildungs‑, Sozial- und Gesundheitswesen. Im hellroten Kreis können die Professionellen sehr nah am Individuum und unter Einbezug des sozialen Umfelds suizidpräventiv agieren. Neben einer effektiven und effizienten Behandlung des beim Individuum aufgetretenen und zu behandelnden (Gesundheits‑)Problems gilt es, generell die psychosoziale Gesundheit des Individuums zu stärken und suizidales Verhalten früh zu erkennen und rechtzeitig zu intervenieren (Früherkennung und Frühintervention); dies unter Berücksichtigung der ganzen Vielfalt und der Ressourcen des:der Jugendlichen, über die verschiedenen Sektoren hinweg und im Rahmen interprofessioneller Zusammenarbeit.

Der hellblaue Kreis steckt den Bereich weiter weg vom Individuum ab. Bei Russel und Fish (2016) wird er als „interpersonaler Kontext“ bezeichnet. Das vorliegende Modell geht einen Schritt weiter, indem es auch Setting-orientierte Maßnahmen der Gesundheitsförderung und Prävention darunter verortet und versteht. Neben Programmen in der Schule, im Sozialwesen, in der Gemeinde oder auch im Kontext des Gesundheitswesens, die gezielt die psychosoziale Gesundheit der Individuen zu stärken und Risikofaktoren (z. B. Schikanierungen in Schulen) über Sensibilisierung und Lebenskompetenzprogramme zu minimieren versuchen, geht es auch um eine grundlegende Veränderung der Strukturen und institutionellen Prozesse (Verhältnisprävention, Setting-orientierte Gesundheitsförderung und Prävention), die einen Kontext schaffen sollen, in dem Suizidversuche und Suizide weniger häufig vorkommen. Eine Schule kann sich etwa über Schulentwicklungsprozesse umfassend gesundheitsförderlich aufstellen, z. B. eingebettet in das WHO-Konzept „Gesundheitsfördernde Schulen“, in der Schweiz etwa im „Schulnetz21“ (www.schulnetz21.ch) verankert. Dies zusätzlich zu LGBTQ+-sensitiven und -spezifischen Maßnahmen (diversitätsgerechte Schulkultur, LGBTQ+-Schulinitiativen, LGBTQ+-Peer-to-peer-Netzwerke), die die Vielfalt innerhalb der LGBTQ+-Jugendlichen berücksichtigen (Intersektionalität!), in andere Sektoren hinein vernetzt sind bzw. unter Einbezug von Professionellen aus dem Sozial- und Gesundheitswesen stattfinden.

Der lila Kreis ist der äußerste Kreis. Er schließt alle Sektoren und Professionellen mit ein. Gemeint sind der soziale und kulturelle Kontext und auch bestehende Normen und Werte in einer Gesellschaft. Normen und Werte schlagen sich in Gesetzen und Politikstrategien nieder, die eher förderlich oder hinderlich für die Lebenslagen und die psychische Gesundheit von LGBTQ+-Jugendlichen sein können. Auch wenn Wirksamkeit hinsichtlich des Einflusses von unterschiedlichen gesellschaftlichen Normen, Systemen und Gesetzen auf die psychische Gesundheit und Suizidrate von LGBTQ+-Personen schwer zu dokumentieren ist, gibt es Anzeichen, dass Einflüsse bestehen [11, 33]. Die Verbesserung der gesetzlichen Gleichstellung von sexuellen und geschlechtlichen Minoritäten und auch eine Entwicklung von Gesellschaften hin zu mehr Offenheit und Akzeptanz gegenüber LGBTQ+-Personen, dem entschiedenen Eingreifen bei Übergriffen etc., ist deshalb ein wichtiger Beitrag zur Suizidprävention. Auch wenn die Professionellen in den Gesundheits‑, Sozial- und Bildungsberufen nicht unmittelbar direkt die gesetzliche Lage und gesellschaftliche Normen und Werte ändern können, so können sie als Expert:innen die Gesellschaft und Politik über die (strukturellen) Hintergründe suizidalen Verhaltens bei LGBTQ+-Personen aufklären und sich für soziale und gesundheitliche Chancengerechtigkeit einsetzen. Denn suizidpräventive Zugriffe innerhalb des hellroten und hellblauen Kreises des vorliegenden Modells reichen nicht aus: Die ganze Gesellschaft und alle Politikbereiche sind gefragt.

## Universelle, selektive und indizierte Suizidprävention

Bekannte universelle, selektive und indizierte Strategien der Suizidprävention können innerhalb des oben vorgestellten Modells verortet und betrieben werden. Auf die Postvention (Interventionen im Umfeld nach erfolgtem Suizid oder Suizidversuch), die auch zur Suizidprävention zu zählen ist [6, 7], wird aus Platzgründen an dieser Stelle nicht eingegangen.

### Universelle Suizidprävention

Universelle Suizidprävention richtet sich an die Gesamtbevölkerung, mit dem Ziel, den Ausbruch einer Ersterkrankung (psychische Störung), eines Suizidversuchs bzw. Suizids zu verhindern [6]. In einer breiteren Definition, angelehnt an das WHO-Verständnis von Gesundheitsförderung, lassen sich darin auch Strategien verorten, die die Lebensqualität und Gesundheit von Individuen und Gruppen generell stärken. Universelle Strategien müssen auch LGBTQ+-Personen miteinschließen, LGBTQ+-inklusiv und LGBTQ+-sensitiv gestaltet sein. Dazu gehört Folgendes:

- die generelle Förderung und Stärkung der psychischen Gesundheit der ganzen Bevölkerung – also auch von LGBTQ+-Personen – mit verhältnis- und verhaltensorientierten Maßnahmen,
- das Schaffen inklusiver Räume im Sozial‑, Bildungs- und Gesundheitswesen, in denen ein gutes (Schul‑)Klima herrscht, keine Stigmatisierung und Diskriminierung stattfindet, auch nicht gegenüber LGBTQ+-Jugendlichen,
- „Gatekeeper“, Personen, die sich im Umfeld von Jugendlichen bewegen (z. B. Ärzt:innen, Sozialarbeitende, Lehrpersonen, Gesundheitspersonal, Eltern, Peers etc.), sollten gestärkt werden, Ressourcen und mögliche Probleme von allen Jugendlichen, also auch LGBTQ+-Jugendlichen, zu erkennen -
- In psychoedukativen Programmen (z. B. in Schulen):
  - … können die psychosozialen Ressourcen von Jugendlichen aktiviert und die psychische Gesundheit gestärkt werden. Dabei bewähren sich insbesondere auch partizipative und Peer-to-peer-Zugänge wie etwa das Beispiel „Kennsch es?“ im Kanton Zug zeigt [29];
  - … können die Jugendlichen lernen, besser zu erkennen, wann es ihnen oder anderen Jugendlichen schlecht geht und wie sie für sich und andere Jugendliche Hilfe holen können;
  - … kann generell eine Kultur des Hilfesuchens und der gegenseitigen sozialen Unterstützung von Jugendlichen etabliert werden.

### Selektive Suizidprävention

Selektive Strategien richten sich an Gruppen mit erhöhtem Suizidrisiko bzw. einem deutlich erhöhten Risiko für suizidales Verhalten. Hierzu gehören auch LGBTQ+-Personen. Die Maßnahmen sind direkt an LGBTQ+-Personen und/oder ihr Umfeld gerichtet oder/und gehen sensibel auf deren Bedürfnisse ein:

- Auf gesellschaftlicher Ebene müssen die Rechte für und die Akzeptanz gegenüber LGBTQ+-Personen gestärkt werden, damit LGBTQ+-Personen im Alltag weniger Hürden und Belastungen vorfinden.
- LGBTQ+-Jugendliche und deren Umfeld (Eltern, Familien, Kolleg:innen) können hinsichtlich psychischer Gesundheit und tragender sozialer Netzwerke (akzeptierendes soziales und familiäres Umfeld) unterstützt werden. Peer-to-peer-Angebote wie etwa LGBTQ+-Jugendgruppen (z. B. Milchjugend, du-bist-du, Transgender Network Switzerland Jugend) oder Eltern, die sich ihre Erfahrung gegenseitig weitergeben (z. B. Angehörigengruppe Transgender Network Switzerland) sind hier von großer Wichtigkeit.
- Alle im vorhergehenden Unterkapitel erwähnten universellen Strategien können auch im engeren Kontext der LGBTQ+-Community angewendet werden; mittels Community-basierter Zugänge, die idealerweise partizipativ gestaltet sind und die Anspruchsgruppen in Teilbereichen ihrer Lebenswelt erreichen (z. B. in LGBTQ+-Onlineforen, Lokalen/Veranstaltungen der LGBTQ+-Community etc.).
- Die Diversitäts- und Handlungskompetenz hinsichtlich LGBTQ+-Themen muss bei Professionellen im Sozial‑, Gesundheits- und Bildungswesen gestärkt werden (Behandlungskompetenz im Gesundheitswesen; Kapazität und Kompetenz z. B. von Schulsozialarbeitenden und Lehrpersonen LGBTQ+-Jugendliche adäquat zu unterstützen etc.).
- Auch mögliche (ausgrenzende) Normen und Mechanismen innerhalb von LGBTQ+-Gruppen müssen kritisch betrachtet und angegangen werden, da auch diese Anpassungsdruck und Probleme bei LGBTQ+-Jugendlichen erzeugen können [15, 19].

### Indizierte Suizidprävention

Indizierte Suizidprävention möchte, so Bronisch [6], suizidales Verhalten „… bei Individuen (verhindern bzw. mindern), die minimale oder aber nachweisbare Anzeichen oder Symptome für ein suizidales Verhalten zeigen“ (z. B. Ritzen; Äußerungen wie „ich kann nicht mehr“, „ich will nicht mehr“ etc.). Sie muss auch an LGBTQ+-Jugendliche gerichtet sein, die Anzeichen suizidalen Verhaltens zeigen.

- Alle Systeme (z. B. Schule, Freizeitbereich, Gesundheits‑, Sozialwesen etc.) müssen darin gestärkt werden, psychosozial belastete und suizidgefährdete LGBTQ+-Jugendliche früh zu erkennen und früh zu intervenieren, ihnen frühzeitig geeignete Hilfen zur Verfügung zu stellen. Die erhöhte Gefährdung von LGBTQ+-Personen für suizidales Verhalten gegenüber der restlichen Bevölkerung muss dabei bekannt sein.
- Auch im engeren Kontext der LGBTQ+-Community selbst, müssen gefährdete LGBTQ+-Personen frühzeitig erkannt werden und es muss eine frühzeitige Intervention erfolgen.

Unter anderem müssen geeignete Hilfeangebote im Umfeld der LGBTQ+-Community bestehen, zu denen Jugendliche Vertrauen haben. Eine Studie in den USA hat etwa gezeigt, dass Krisendienste innerhalb der LGBTQ+-Community gegenüber allgemeinen Angeboten eine wichtige Funktion einnehmen und genutzt werden [13]. Wichtig ist, dass solche (LGBTQ+-spezifischen) Dienste nicht einfach ehrenamtlich von Engagierten getragen werden, sondern auch staatlich finanziert sind und eine entsprechende Anerkennung und strukturelle Verankerung erhalten.

## Schlussfolgerungen

Die LGBTQ+-Jugendlichen müssen sowohl in universellen, selektiven wie auch indizierten Strategien der Suizidprävention konsequent mitbedacht werden. Die ganze Gesellschaft und alle Professionen können einen Beitrag zur Reduktion der Suizidversuche und Suizide bei diesen leisten. Wichtig zu vermerken ist: International fehlt es an suizidpräventiven Zugängen für LGBTQ+-Jugendliche, entsprechend gibt es auch wenig Hinweise hinsichtlich der Wirkungen solcher Zugänge. Das im Artikel vorgeschlagene Modell soll die dringend benötigte Konzeption, Implementation und Evaluation von suizidpräventiven Maßnahmen für LGBTQ+-Jugendliche beschleunigen. Es muss auf der Basis künftiger empirischer Ergebnisse und systematischer Literaturreviews kritisch geprüft, ergänzt oder gar verworfen werden. Der Autor stellt an dieser Stelle lediglich ein noch sehr vorläufiges Modell zur Debatte, das der Public-Health-Praxis einen Rahmen an die Hand gibt, um suizidpräventive Maßnahmen auf verschiedenen Ebenen zu verorten, zu planen und durchzuführen, dies intersektional und über verschiedene Professionen und Sektoren hinweg. Weiterhin besteht ein Bedarf an anwendungsorientierter Forschung, die noch präziser die genauen Umstände und Hintergründe von Suizidversuchen von LGBTQ+-Jugendlichen festmacht und basierend darauf die Weiterentwicklung suizidpräventiver Zugänge für diese Jugendlichen orientieren kann.
